# Rab-mediated vesicular transport is required for neuronal positioning in the developing *Drosophila *visual system

**DOI:** 10.1186/1756-6606-3-19

**Published:** 2010-06-11

**Authors:** Tarek Houalla, Lei Shi, Donald J van Meyel, Yong Rao

**Affiliations:** 1 McGill Centre for Research in Neuroscience, Department of Neurology and Neurosurgery, Department of Medicine, McGill University Health Centre, 1650 Cedar Avenue, Montreal, Quebec H3G 1A4, Canada

## Abstract

**Background:**

The establishment of tissue architecture in the nervous system requires the proper migration and positioning of newly born neurons during embryonic development. Defects in nuclear translocation, a key process in neuronal positioning, are associated with brain diseases such as lissencephaly in humans. Accumulated evidence suggests that the molecular mechanisms controlling neuronal movement are conserved throughout evolution. While the initial events of neuronal migration have been extensively studied, less is known about the molecular details underlying the establishment of neuronal architecture after initial migration.

**Results:**

In a search for novel players in the control of photoreceptor (R cell) positioning in the developing fly visual system, we found that misexpression of the RabGAP RN-Tre disrupted the apical localization of R-cell nuclei. RN-Tre interacts with Rab5 and Rab11 in the fly eye. Genetic analysis shows that Rab5, Shi and Rab11 are required for maintaining apical localization of R-cell nuclei.

**Conclusions:**

We propose that Rab5, Shi and Rab11 function together in a vesicular transport pathway for regulating R-cell positioning in the developing eye.

## Background

Normal brain function requires the proper formation of neuronal architecture during development. After birth, many neuronal precursor cells need to undergo long-distance migration to reach their destination [1-3]. Upon reaching correct brain regions, neuronal precursor cells undergo further morphological changes to organize themselves into discrete layers or clusters, differentiate, and elaborate precise connections with their target cells [4]. Neuronal movement occurs in several steps. First, a neuron extends a leading process or neurite that explores the environment for navigational cues [5-7]. Then, following the consolidation of the leading process, the nucleus is translocated into the leading process (i.e. nucleokinesis), which is closely followed by the retraction of the trailing process [8-11].

Accumulated evidence support the importance of nuclear translocation in neuronal positioning during development. For instance, defects in nuclear translocation have been suggested to be responsible for the failure of newly born neurons to migrate to the cortical surface during development, leading to abnormal brain structures and mental retardation in Lissencephaly patients [12,13]. The molecular mechanism regulating nuclear translocation in neuronal migration appears to be conserved throughout evolution. Lis1, the corresponding gene of Lissencephaly [14,15], has been shown to be evolutionarily conserved, whose orthologues have been identified in organisms like aspergillus nidulans [16] and *Drosophila *[17,18]. Lis-1 interacts with components of the evolutionarily conserved migratory machinery including microtubules [19], the minus-end directed motor protein dynein [16-18] as well as several dynein-associated proteins such as NUDE [20], NUDC [21] and NUDL [22,23].

The translocation of photoreceptor (R cell) nuclei in the developing *Drosophila *visual system has been proven to be an excellent model system for genetic dissection of the general mechanisms controlling neuronal positioning during development [18,24-26]. The development of the *Drosophila *adult compound eye begins at the third-instar larval stage, when precursor cells differentiate into R cells in the eye-imaginal disc. The eye disc has a single-layered epithelial structure in which cells are continuous from the apical surface to the basal surface, while the nuclei of the cells undergo dynamic translocation. The development of eye disc is marked by the posterior-to-anterior movement of the morphogenetic furrow across the eye disc. The movement of the furrow involves cycles of dynamic cell movement along the apical-basal axis. Initially, anterior precursor cell bodies and nuclei translocate basally, leading to the formation of the furrow. After exiting the furrow, precursor cells begin to differentiate into R cells sequentially, and the nuclei of differentiating R cells translocate apically [27]. Genetic dissection of R-cell nuclear translocation has shown that this process utilizes evolutionarily conserved mechanisms. For instance, DLis, the *Drosophila *homolog of human Lis-1, is required for the apical translocation of R-cell nuclei [18]. Apical translocation of R-cell nuclei also requires evolutionarily conserved genes such as dynactin [28,29], klarsicht [24,30], nuclear lamin [31], Bicaudal-D (Bic-D) [18,25], Misshapen (Msn) [25], and Klaroid [26].

While much is known about the control of the initial basal-to-apical translocation of R-cell nuclei, less is known about how apical localization of R-cell nuclei is maintained during development. In a search for novel players in the control of R-cell positioning, we found that misexpression of the fly homolog of the mammalian Rab GTPase-activating-protein (GAP) RN-Tre caused a failure of R-cell nuclei to maintain their apical localization in the developing eye. Mammalian cell culture studies show that RN-Tre can negatively regulate Rab proteins [32,33]. Since Rabs are important regulators of intracellular vesicular transport [34], we set out to determine the nature of vesicular transport that is required for the maintenance of R-cell apical localization. Our results support the involvement of a Rab5-Shibire/dynamin-Rab11-dependent vesicular transport pathway in R-cell positioning.

## Methods

### Genetics

*GMR-GAL4*, *EyGAL4 *and *UY333 *lines were provided by the Bloomington *Drosophila *stock center. GS lines containing bi-directional UAS elements were generated by remobilizing the P{GS1} P element [35,36]. *Rab5*^2 ^, FRT 40 was provided by D.Bilder. *Rab5S43N *was provided by M. Gonzalez-Gaitan. UAS-*shi*^1 ^was provided by Y. Zhong. *Rab11*^ex2 ^*and Rab11*^ex1 ^were provided by R.S. Cohen. UAS-*Rab11-RNAi *was provided by D.F. Ready. *Rab6*^D23D ^was provided by A. Guichet. *Rab5*^2 ^and *Rab11*^ex2 ^mutant tissues were generated in the eye using the *eyFLP-FRT *system [37]. To label *Rab11*^ex2 ^mutant clones, *eyFLP*; FRT82B, GMR-*myr.GFP *flies were crossed with FRT82B, *Rab11*^ex2 ^flies. The dosage of *Rab5*, *Rab6 *or *Rab11 *was reduced by 50% in flies overexpressing RN-tre by crossing female flies *yw; Cyo*/UAS-*RN-tre*; long-*GMR*-*GAL4*/*TM3, Tb *with male flies *w; Cyo*/*Rab5*^2^, *yw; Cyo*/*Rab6*^D23D^, or *Rab11*^ex2^/*TM3, Sb*, respectively. The full-length *RN-tre *cDNA (LD38355) was subcloned into the pUAST vector. The resulting construct was used to generate UAS-*RN-tre *transgenic lines (BestGene Inc., CA).

### Histology and Immunohistochemistry

Eye discs were dissected from third-instar larvae in ice cold phosphate-buffered saline (PBS, pH 7.0), and fixed in 2% paraformaldehyde in PBL (0.075 M lysine, 0.1 M sodium phosphate buffer, pH 7.4) for 45 minutes at room temperature. Eye discs were washed three times in PBT (0.5% Triton-X-100 in PBS) for 10 minutes, and then blocked in 10% normal goat serum (NGS) in PBT for 1 hour at room temperature. Eye discs were subsequently incubated with primary antibody overnight at 4°C, washed three times in PBT for 10 minutes each, and then incubated with secondary antibody for 1 hour at room temperature. The discs were then washed three times in PBT, and once in PBS for 10 minutes each, and then incubated for 10 minutes in *SlowFade *Gold antifade reagent (Invitrogen) before mounting. Primary antibodies used are as follows: Chaoptin (24B10) (Developmental Studies Hybridoma Bank or DSHB, 1:200 dilution); Elav (DSHB, 1:500 dilution); Rab11 (provided by D.F.Ready, 1:1000 dilution); Cy2 AffiniPure Goat Anti-Horseradish Peroxidase (Jackson ImmunoResearch, 1:30 dilution). Secondary antibodies include antibodies from Jackson Immunochemicals: Texas-red-or FITC-conjugated goat anti-rabbit or anti-mouse antibodies (1:200 dilution); and antibodies from Molecular Probes: Alexa Fluor 488 goat anti-mouse and goat anti-rabbit IgG, Alexa Fluor 568 goat anti-rat and goat anti-rabbit IgG, and Alexa Fluor 647 goat anti-mouse IgG. All Alexa antibodies were used at 1:200 dilution. High-resolution fluorescence imaging system (Canberra Packard) was used to capture Z-series images, which were analyzed by 2D Deconvolution using the MetaMorph imaging software (Universal Imaging, Brandywine, PA). Longitudinal optic sectioning of eye discs was performed using confocal microscopy

Plastic sectioning of the eye disc stained with anti-Elav antibody was performed as described previously [25].

### EMS mutagenesis

Homozygous *GSd427 *males aged 3-5 days were starved for 6 hours and fed with 2.5 mM ethylmethanesulfonate (EMS) in 1% sucrose overnight. The males were then crossed with females carrying *GMR*-*GAL4 *for 24 hours in fresh bottles, and were transferred to new bottles every 12 hours for 4 days. The adult progeny (*GSd427*/*GMR*-*GAL4*) were then collected and screened for the suppression of the reduced eye phenotype under dissection microscope. *GSd427 *mutant chromosomes showing suppression were balanced over *CyO*.

Homozygous mutants for the *GSV *allele of *GSd427 *isolated from the screen were viable. The coding region of the *RN-tre *gene in homozygous *GSV *mutants was amplified by PCR and sequenced, which was compared to that in wild type and *GSd427*.

## Results

### A mis-localization phenotype induced by misexpression of genes in R cells

To search for genes required for R-cell development in the fly visual system, we crossed a collection of UAS-containing P-element insertion lines (i.e. GS lines) [35,36] with the eye-specific *GMR-GAL4 *line to drive the expression of genes adjacent to the insertion site in R cells. Since *GMR-GAL4 *is expressed after cells exit the morphogenetic furrow, this allowed us to specifically assess the effect of gene misexpression on R-cell development after cells exit the furrow. R-cell nuclei were visualized using an antibody recognizing the neuronal-specific nuclear protein Elav.

**Figure 1:**
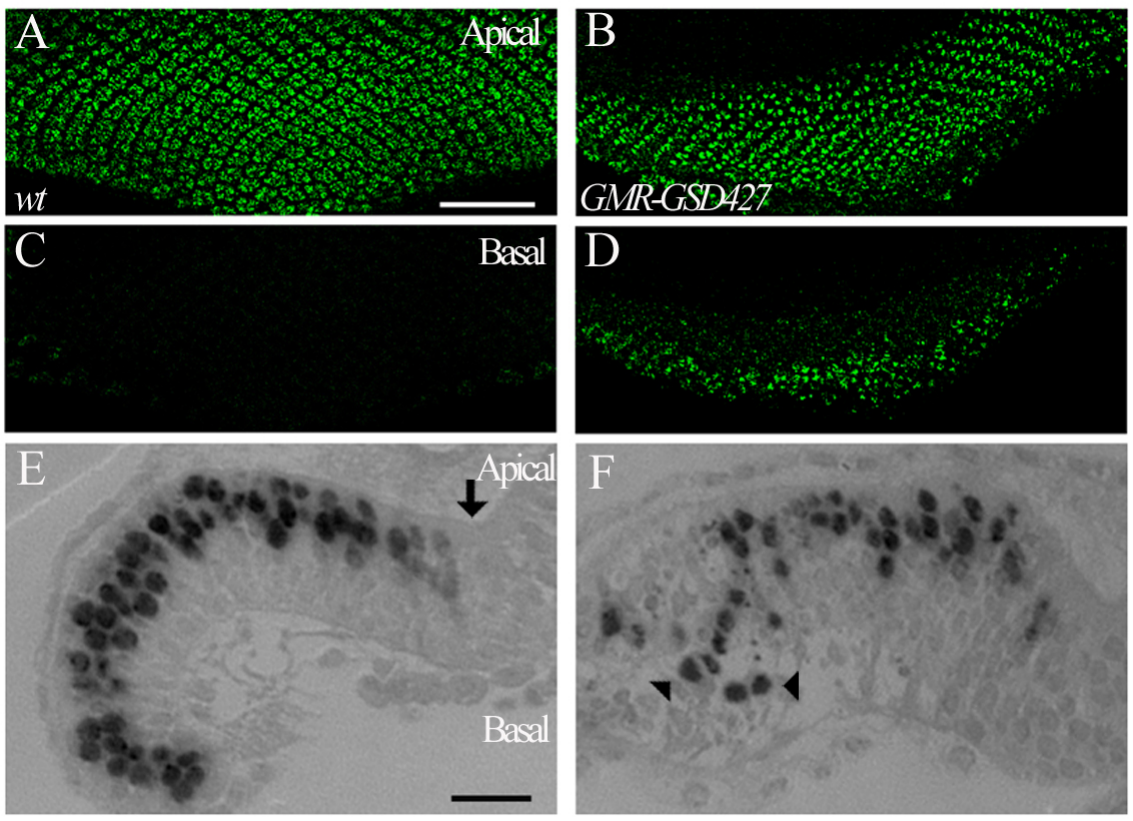
***GSd427 *driven by *GMR-GAL4 *caused defects in R-cell localization**. (A-D) Apical (A and B) and basal (C and D) view of third-instar larval eye discs stained with anti-Elav. In wild type (A), R-cell bodies and nuclei are located at the apical region of the eye disc, and are absent at the basal region (C). The distance between the sections shown in A and C is ~12 μm. In larvae carrying *GSd427 *under control of *GMR-GAL4 *(B and D), many R-cell nuclei were mis-localized to the basal region (D). The distance between the sections shown in B and D is ~13 μm. (E and F) Longitudinal plastic sections of eye discs stained with anti-Elav (1 μm thick). Apical is up and basal is down. (E) In wild type, R-cell nuclei tanslocate to the apical region in the region posterior to the morphogenetic furrow (arrow). (F) In *GSd427 *larvae, many R-cell nuclei (arrowheads) were mis-localized to the basal region (~27.1% of ommatidia, n = 8 eye discs). Note that the majority of R-cell nuclei were able to translocate to the apical region at earlier stage (i.e. R cells located close to the furrow). Scale bars: A-D, 50 μm; E and F, 10 μm.

We found that one P-element insertion *GSd427 *displayed a severe defect in R-cell positioning. In wild type (Figure 1A,C and 1E), after exiting the furrow, precursor cells differentiate sequentially into R cells, and R-cell nuclei translocate from the basal region to the apical region, and maintain their apical localization throughout development. When gene/genes adjacent to the insertion *GSd427 *were misexpressed with *GMR-GAL4*, we found that although the initial translocation occurred normally as the nuclei of newly recruited R cells in the posterior region close to the furrow (i.e. within the distance of 6-7 rows of R-cell clusters) were able to translocate to the apical region, many of them failed to maintain their apical localization at later stage (i.e. R cells located at more posterior region) and appeared at the basal region instead (Figure 1B,D and 1F).

**Figure 2:**
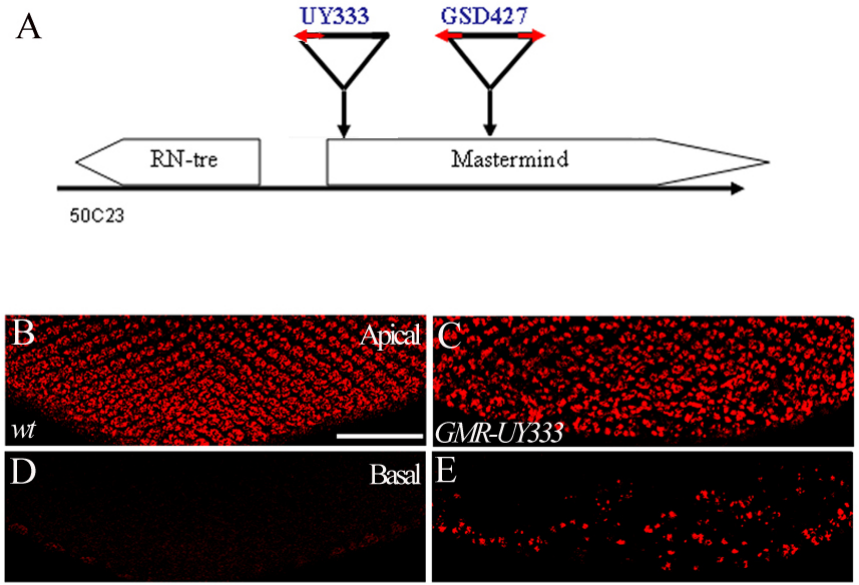
**Uni-directional misexpression driven by the combination of *UY333 *and *GMR-GAL4 *caused R-cell defects identical to the *GSd427 *phenotype**. (A) Relative positions of *GSd427 *and *UY333 *P-element insertion sites in the genome. Both P elements are inserted into the cytological region 50C23. *GSd427 *is inserted in a region ~5.5 kb upstream of *RN-Tre *and ~5 kb downstream of the *mam *transcriptional initiation site, while *UY333 *is inserted into a site ~588 base pairs upstream of *RN-tre*. *GSd427 *carries bi-directional UAS elements (red arrows) that can potentially drive gene transcription in both plus- and minus directions, while *UY333 *carries one UAS element that can drive gene expression only at the direction of *RN-tre*. (B-E) Apical (B and C) and basal (D and E) view of third-instar larval eye discs stained with anti-Elav antibody. (B and D) Apical (B) and basal (D) optic sections of wild-type eye discs. The distance between the sections shown in B and D is ~13 mm. (C and E) Apical (C) and basal (E) optic sections of *GMR-GAL4-UY333 *eye discs. Many R-cell nuclei were mis-localized to the basal region (E) (~34%, n = 1026 ommatidia in 6 eye discs). The distance between the sections shown in C and E is ~13 μm. Scale bar: B-E, 50 μm.

***RN-tre *is the corresponding gene of the *GSd427 *misexpression phenotype ***GSd427 *is inserted into a cytological region at 50C23 on the second chromosome. Molecular mapping data showed that *GSd427 *is inserted within the *mastermind *(*mam*) gene, ~5.0 kb downstream of the transcription initiation site of *mam*, and ~5.5 kb upstream of the *RN-tre *gene (Figure 2A). Since the P-element insertion in *GSd427 *contains bidirectional UAS elements, it can potentially drive gene misexpression in the direction of *mam *or *RN-tre*.

To determine if *mam *or *RN-tre *was responsible for the *GSd427 *phenotype, we took the advantage of a P-element insertion called *UY333 *located at a site ~588 bp upstream of the *RN-tre *transcriptional initiation site and ~100 bp downstream of the *mam *transcriptional initiation site (Figure 2A) [38]. Since *UY333 *contains a single UAS element, it can only drive gene misexpression in the direction of *RN-tre*. *UY333 *driven by *GMR-GAL4 *caused a R-cell mis-localization defect (Figure 2C and 2E) that was indistinguishable from that in *GSd427 *misexpression mutants (Figure 1). This result suggests that the gene corresponding to the *GSd427 *gain-of-function phenotype is *RN-tre*, or other genes located at the *RN-tre *side of the insertion site.

**Figure 3:**
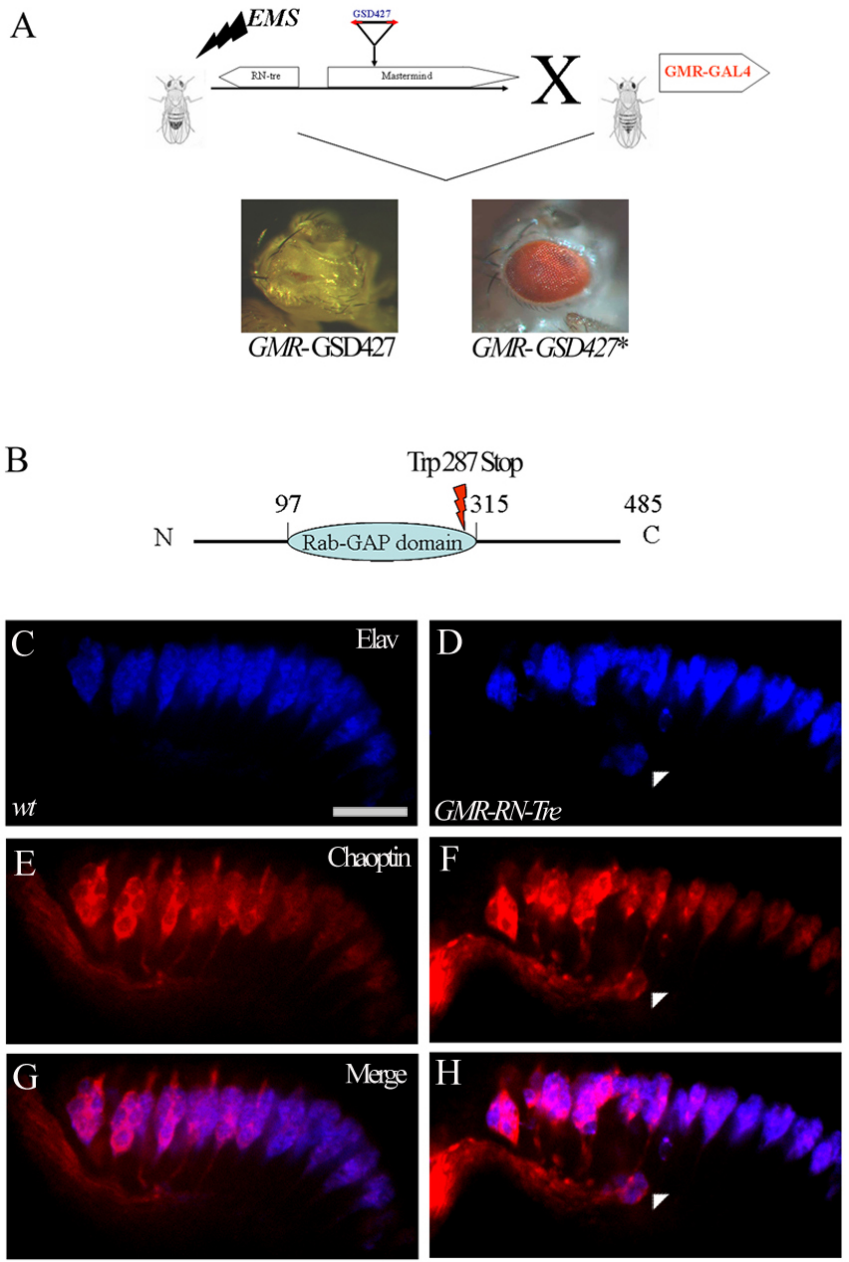
***RN-tre *is the corresponding gene of the *GSd427 *and *UY333 *misexpression phenotype**. (A) An EMS mutagenesis screen for identifying the suppressors of the *GSd427 *gain-of-function eye phenotype. Males carrying the *GSd427*-containing chromosome were mutagenized by EMS ingestion and then crossed with females carrying *GMR-GAL4*. The F1 progeny were screened for the suppression of the reduced eye phenotype. (B) Sequencing of the *RN-tre *gene in one suppressor (GSV) allele reveals a non-sense point mutation at nucleotide 1119 that converts the codon for Trp into a stop codon at amino-acid 287. (C-H) Longitudinal optic sectioning of third-instar eye discs double-stained with anti-Elav (blue) and MAb 24B10 (Chaoptin) (red) antibodies to visualize R-cell nuclei and R-cell surface, respectively. (C, E and G) Wild-type eye discs show apical localization of R cells. (D, F and H) Misexpression of UAS-*RN-tre *under control of *GMR-GAL4 *induced basal localization of R cells (D, F, H, arrowheads) (~32%, n = 1008 ommatidia in 6 eye discs). Scale bar: 10 μm.

To further determine the identity of the gene whose misexpression caused the R-cell mis-localization phenotype, we performed an EMS mutagenesis screen to isolate mutations on the *GSd427*-containing chromosome that suppress the *GSd427 *gain-of-function phenotype. We expected that some suppressor mutations would disrupt the function of the corresponding gene of the *GSd427 *misexpression phenotype. This approach has two advantages. First, it allows us to identify the corresponding gene. Second, it has the potential to generate strong loss-of-function mutant alleles in that gene. Since *GSd427 *under control of *GMR-GAL4 *caused a severe adult rough eye phenotype, we were able to perform a large-scale genetic screen to isolate mutations on the *GSd427*-carrying chromosome that suppress the rough eye phenotype (Figure 3A). From ~26,000 mutated *GSd427*-containing chromosomes, we established two lines with mutations that suppressed the rough eye phenotype associated with *GSd427 *misexpression. As predicted, both mutations also suppressed the *GSd427 *R-cell mis-positioning phenotype (data not shown). To determine the identity of the corresponding gene, we sequenced the mutant chromosome and identified a non-sense mutation (i.e. *RN-tre*^GSV ^) in the *RN-tre *gene that converts a codon for Trp287 into a stop codon. This mutation is predicted to generate a truncated protein missing part of the Rab-GAP domain and the complete C-terminal regulatory region (Figure 3B).

While results from above analysis are consistent with that *RN-tre *is the corresponding gene whose overexpression caused the observed phenotype, it remains possible that haploinsufficiency of *mam *contributed to the phenotype as P element in both *GSd427 *and *UY333 *inserted into the *mam *gene. To further address this, we examined if misexpression of a *RN-tre *transgene in R cells is sufficient for inducing mis-localization phenotype. Indeed, we found that misexpression of *RN-tre *under control of *GMR-GAL4 *caused a *GSd427*- and *UY333*-like mis-localization phenotype (Figure 3D). We conclude that *RN-tre *is the gene whose misexpression caused the R-cell mis-localization phenotype.

### R-cell positioning occurs normally in *RN-tre *mutants

**Figure 4:**
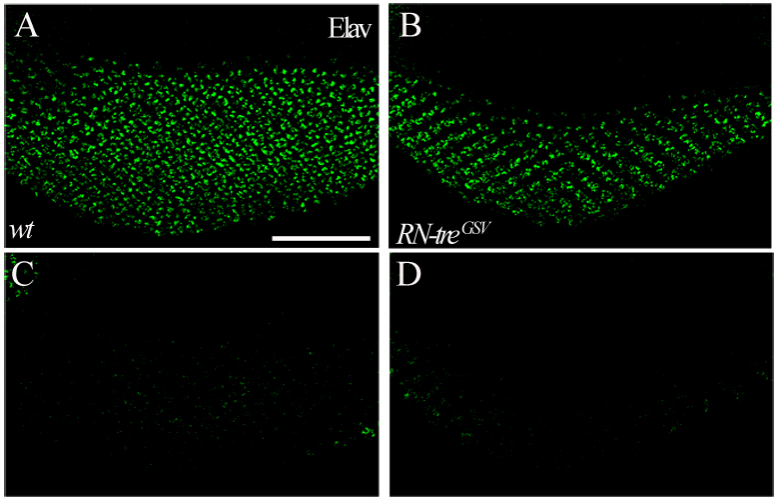
**Loss of *RN-tre *did not affect R-cell nuclear positioning**. (A-D) Apical (A and B) and basal (C and D) view of third-instar larval eye discs stained with anti-Elav. (A and C) Wild type. The distance between the sections shown in A and C is ~16 μm. (B and D) In *RN-Tre*^GSV ^mutants, R-cell nuclei appeared normally in the apical region (B) and were not found in the basal region (D) (n = 13 eye discs). The distance between the sections shown in B and D is ~16 μm. Scale bar: 50 μm.

To determine if RN-Tre is required for R-cell morphogenesis in the developing fly visual system, we performed loss-of-function analysis of the *RN-tre *mutants generated in our suppression screen. Third-instar larvae homozygous for the *RN-tre*^GSV ^allele were examined for potential defects in R-cell nuclear translocation. However, no defect was observed (Figure 4). Thus, it remains unclear if endogenous RN-Tre plays a role in R-cell morphogenesis during development.

### RN-Tre interacts with Rab5 and Rab11 in the eye

**Figure 5:**
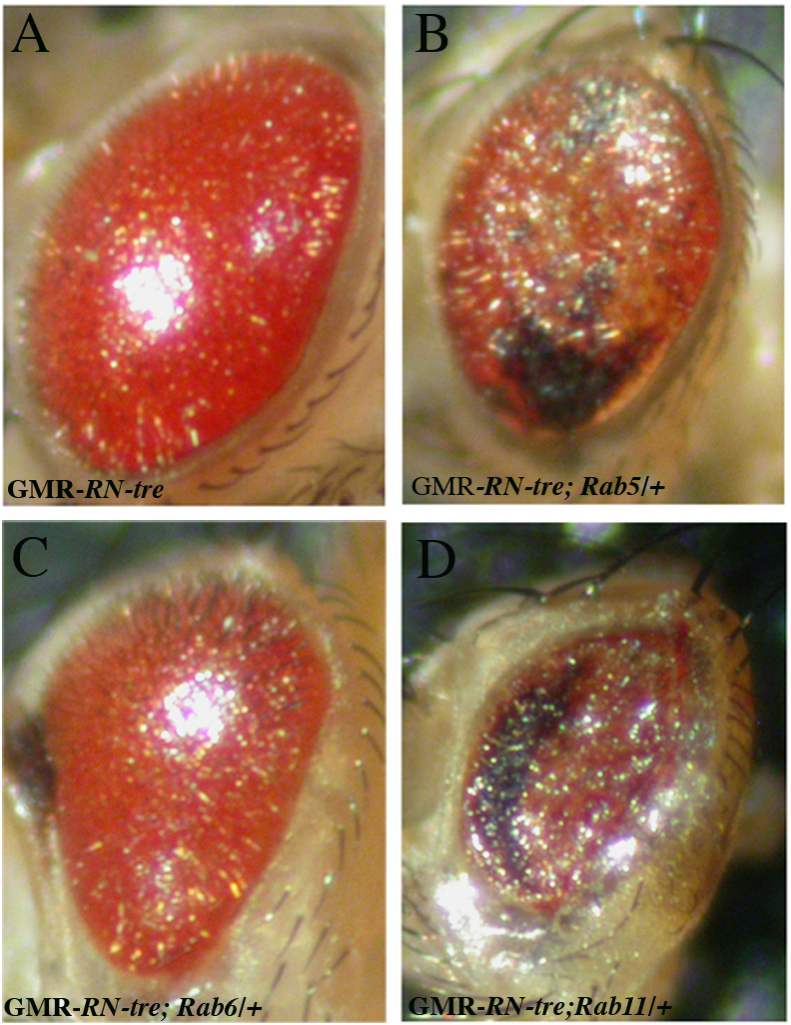
***Rab5 *and *Rab11 *mutations enhanced the *RN-tre *overexpression eye phenotype**. (A) Ovexpression of *RN-tre *under control of the long*GMR-GAL4 *driver caused a mild rough eye phenotype. The eye appeared glassy and smooth. Genotype: UAS-*RN-tre*/+; long*GMR-GAL4*/+. (B) Reducing the dosage of *Rab5 *by 50% enhanced the *RN-tre *overexpression eye phenotype. The eye became flat and displayed a necrotic phenotype. Genotype: UAS-*RN-tre*/*Rab5*^2^; long*GMR-GAL4*/+. (C) Reducing the dosage of *Rab6 *did not enhance the *RN-tre *overexpression eye phenotype. Genotype: UAS-*RN-tre*/*Rab6*^D23D^; long*GMR-GAL4*/+. (D) Reducing the dosage of *Rab11 *also significantly enhanced the RN-tre overexpression eye phenotype. Genotype: UAS-*RN-tre*/*+*; long*GMR-GAL4*/*Rab11*^ex2^.

Since mammalian RN-Tre has been shown to possess GAP activity and negatively regulate the activity of certain Rabs in cultured cells [32,33,39], we speculated that the *RN-tre *misexpression phenotype reflects a requirement for Rab-mediated vesicular transport in R-cell positioning during development. To assess this, we examined the potential genetic interaction between RN-Tre and several Rab family proteins. We used the long*GMR-GAL4 *driver to overexpress UAS-*RN-tre *in the fly eye. This eye-specific driver is more specific to photoreceptor cell than the regular *GMR-GAL4 *driver [40](see previous section). Overexpression of UAS-*RN-tre *under control of the long*GMR-GAL4 *driver caused a rough eye phenotype that is much milder than that induced by *GMR-GAL4 *(Compare Figure 5A to Figure 3A; 100%, n > 30 eyes). We reasoned that if this rough eye phenotype is due to the negative regulation of the activity of some Rab proteins by overexpression of *RN-tre*, reducing the dosage of such Rab proteins would enhance the rough eye phenotype. To test this, we examined the effect of reducing the dosage of *Rab5*, *Rab6 *or *Rab11 *by 50% on the *RN-tre*-induced eye phenotype. We found that reducing the dosage of *Rab5 *(52%, n = 76 eyes, Figure 5B) or *Rab11 *(90%, n = 20 eyes, Figure 5D) significantly enhanced the *RN-tre*-induced rough eye phenotype, whereas no such enhancement was observed when the dosage of *Rab6 *was reduced (0%, n = 24 eyes, Figure 5C). This result suggests that overexpression of RN-tre negatively regulates the activity of Rab5 and Rab11 in the fly eye.

### Rab5 is required for apical localization of R-cell nuclei

Rab5 is a key regulator of protein internalization, early endocytic vesicular transport and the fusion of endocytic vesicles with early endosomes [41,42]. To address the possibility that the RN-Tre misexpression phenotype reflects an active role for Rab5 in the control of R-cell apical localization, we performed loss-of-function analysis to determine if mutations in *Rab5 *affect R-cell nuclear positioning. Large patches of mutant clones homozygous for a *Rab5 *null allele (i.e.*Rab5*^2^) were generated in the eye using *eyFLP/FRT*-based mitotic recombination [37]. However, we were unable to analyze R-cell positioning in homozygous null tissues as complete loss of Rab5 prevented R-cell differentiation and led to the overgrowth of eye-imaginal discs (data not shown), which is consistent with the previous observation by Bilder and colleagues [43].

**Figure 6:**
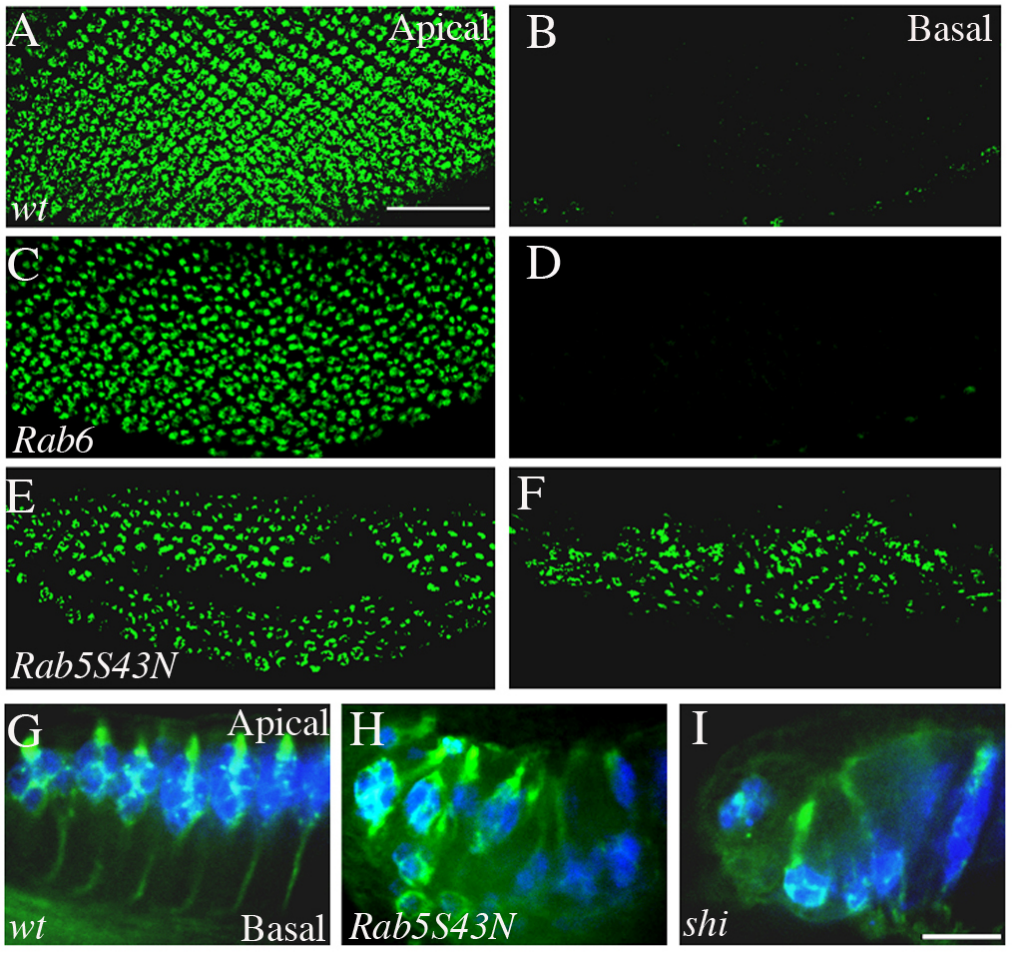
**Rab5 and Shi are required for apical localization of R-cell nuclei in the eye disc**. (A-F) Apical (A, C and E) and basal (B, D and F) view of third-instar larval eye discs stained with anti-Elav antibody. The distance between apical and basal sections is ~15 μm. (A and B) In wild type, R-cell nuclei are localized to the apical region (A) and thus are not present in the basal region (B). (C and D) In a *Rab6 *null (*Rab6*^D23D^) mutant eye disc, R-cell nuclei appeared normally at the apical region (C). (E and F) In an eye disc expressing the dominant-negative form of Rab5 (Rab5S43N), many R-cell nuclei were missing in the apical region (E) and mis-localized to the basal region (F). (G-I) Longitudinal optic sectioning of third-instar eye discs double-stained with anti-Elav (blue) and anti-HRP (green) to visualize R-cell nuclei and R-cell surface, respectively. (G) Wild type. (H) In eye discs expressing Rab5S43N, ~27% ommatidia contained mis-localized R-cell nuclei (n = 5 eye discs). (I) Eye discs expressing the dominant-negative form of shi (i.e. *shi*^*t*^^s1^) displayed a R-cell mis-localization phenotype (~34% ommatidia, n = 7 eye discs). Scale bars: A-F, 50 μm; G-I, 10 μm.

To circumvent this problem, we took an alternative approach. A dominant-negative form of Rab5 (Rab5S43N) was expressed in the posterior region of the third-instar larval eye disc under control of *GMR-GAL4*. Eye discs expressing this Rab5S43N displayed normal morphology, and differentiating R cells in the eye disc also expressed normal R-cell developmental markers (Figure 6E,F and 6H). However, we found that many R-cell nuclei failed to maintain their apical localization (Figure 6E) and instead mis-localized basally (Figure 6F and 6H). By contrast, loss of Rab6, a key regulator of ER-to-Golgi vesicular trafficking [44,45], has no effect on R-cell nuclear positioning (Figure 6C and 6D).

### Shibire/dynamin is also required for R-cell apical localization

Above results suggest that the Rab5-mediated early endocytic pathway is required for R-cell apical localization. If so, one may predict that mutations in other genes involved in early endocytic trafficking should also affect R-cell positioning. To address this possibility, we tested whether loss of Shibire (Shi), the fly homolog of dynamin that is another key regulator of endocytosis, affects R-cell nuclear localization.

We used a temperature-sensitive, dominant-negative form of *shi *(UAS-*shi*^ts1^) to examine the effect of reducing the activity of Shi on R-cell positioning. It has been shown previously that at the restrictive temperature (i.e. 32°C) *shi*^ts1 ^could completely block endocytosis (e.g. [46,47]). We used *GMR-GAL4 *to express UAS- *shi*^ts1 ^specifically in the posterior region of the third-instar larval eye disc. R-cell localization appeared normal at permissive temperature (i.e. 18°C) (data not shown). In contrast, when larvae were reared at restrictive temperature, they displayed a severe R-cell nuclear mis-localization phenotype (Figure 6I) that was indistinguishable from that in larvae expressing the dominant-negative form of Rab5S43N (Figure 6H). Together, these results point to a key role for the early endocytic pathway in maintaining apical localization of R- cell nuclei in the developing eye disc.

### Rab11-mediated recycling is also required for R-cell positioning

One possible explanation for the Rab5- and Shi mis-localization phenotype is that endocytosis of certain membrane proteins maintains the apical localization of R-cell nuclei during development. Alternatively or additionally, Rab5- and Shi-mediated endocytosis may be necessary for retargeting these membrane proteins to specific subcellular locations by a Rab-regulated vesicular recycling pathway. The latter hypothesis is supported by that reducing the dosage of *Rab11 *also significantly enhanced the *RN-tre*-induced rough eye phenotype (see Figure 5). To further address this, we tested if loss of Rab11, an evolutionarily conserved key regulator of vesicular recycling [48,49], affected R-cell positioning.

**Figure 7:**
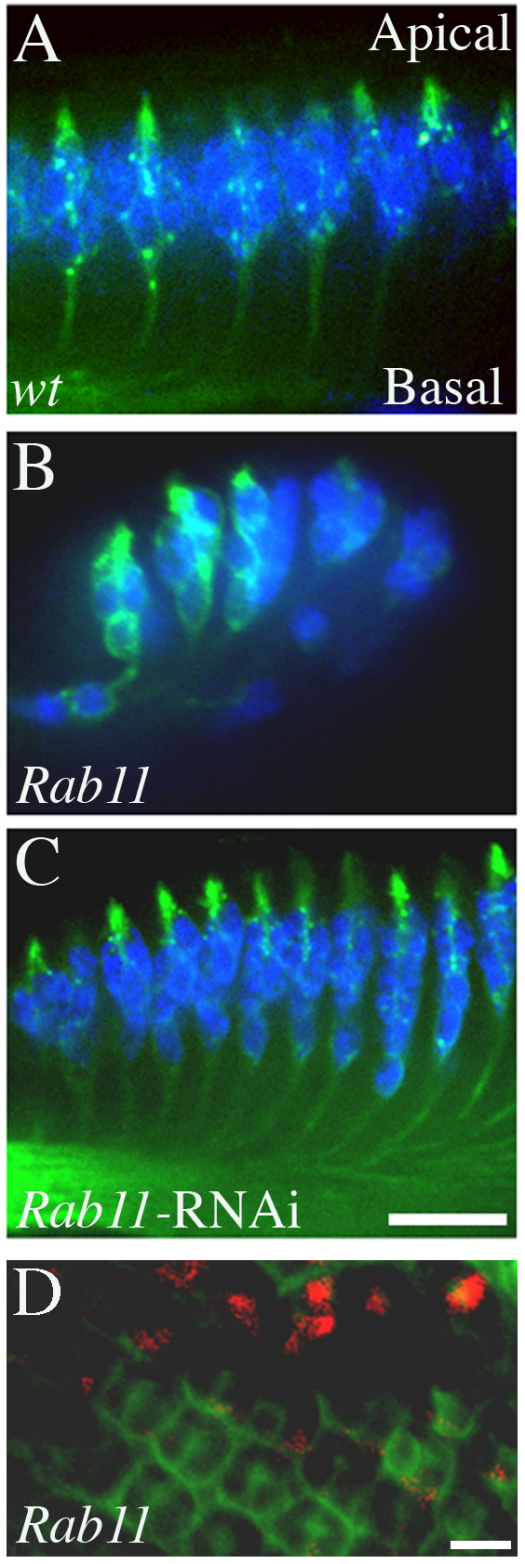
**Rab11 is also required for R-cell positioning**. (A-C) Longitudal optic sectioning of third-instar larval eye discs double-stained with anti-Elav (blue) and anti-HRP (green) to visualize R-cell nuclei and R-cell surface, respectively. (A) In wild type, R-cell bodies localizes to the apical region, while their apical processes attach to the apical surface and their axons project basally. (B) The generation of clones of *Rab11*^ex2 ^mutant cells caused R-cell nuclei to be mis-localized at the basal region. (C) In eye discs expressing a UAS-*Rab11-RNAi *transgene, R cells did not appear to be tightly associated clusters at the apical region and instead formed a long stretch along the apical-basal axis (20 out of 41 eye discs examined). Note that R-cell processes still attached to the apical surface in *Rab11 *mutants (B and C). (D) Basal localization of *Rab11 *mutant nuclei in *Rab11*^ex2 ^mosaic eye disc. GFP (green) was used to label wild-type or *Rab11 *heterozygous ommatidia in *Rab11 *mosaic eyes. R-cell nuclei at the basal region were visualized with anti-Elav staining (red). In GFP-positive ommatidia, no R-cell nuclei were observed in the basal region. In the basal region of homozygous *Rab11 *mutant ommatidia or mosaic ommatida (GFP negative), mis-localized R-cell nuclei (red) were observed. Scale bar: A-C,10 μm; D, 5 μm.

Tissues homozygous for a strong *Rab11 *loss-of-function allele *Rab11*^ex2 ^were generated in the eye using *eyFLP/FRT*-induced mitotic recombination [37]. While mutant R-cell clusters still projected processes that attached to the apical surface and correctly sent axons basally, we found that many R-cell nuclei appeared abnormally at the basal side (Figure 7B and 7D).

We also examined if silencing *Rab11 *by RNA interference (RNAi) approach caused a similar R-cell mis-localization phenotype. We expressed a UAS-*Rab11-RNAi *transgene specifically in the eye, which has been shown previously to significantly reduce the level of Rab11 in R cells [50]. Indeed, we found that reducing the level of Rab11 with RNAi also caused a failure for R-cell nuclei to maintain their apical localization (Figure 7C). Instead of forming a tightly associated R-cell cluster in the apical region, as is observed in wild type (Figure 7A), R-cell clusters in *Rab11 RNAi *individuals extended in a long stretch with the distribution of R-cell nuclei along the entire apical-basal axis (Figure 7C).

That loss of Rab11 caused a Rab5- and Shi-like R-cell mis-localization phenotype suggests that Rab5, Shi and Rab11 function together in vesicular transport to maintain apical localization of R cells during development.

## Discussion

The movement of R-cell nuclei along the apical-basal axis in the developing fly visual system displays features very similar to the somal translocation of neurons from the ventricular zone to the cortical plate during the development of the mammalian cerebral cortex [51,52]. That both R-cell movement and cortical neuronal migration require the function of DLis/Lis1 [14,18] support the evolutionary conservation of the molecular mechanism controlling neuronal positioning. In a search for novel regulators of R-cell translocation, we found that misexpression of the RabGAP RN-Tre caused a failure for R-cell nuclei to maintain their apical localization, suggesting the requirement of Rab-mediated vesicular transport in R-cell positioning. *RN-tre *displayed dosage-sensitive interactions with *Rab5 *or *Rab11 *in the fly eye, and genetic analysis revealed an essential role for Rab5, Shi and Rab11 in R-cell apical localization. These results support that Rab5, Shi and Rab11 function together in a vesicular transport pathway to regulate R-cell positioning during development.

RN-Tre, like most of RabGAPs, contains a Tre2/Bub2/Cdc16 (TBC) domain that accelerates the GTPase activity of Rabs with an Arg-Glu dual-finger mechanism [53]. Mammalian RN-Tre was originally identified by Di Fiore and colleagues as a binding partner of Eps8, a substrate of the epidermal growth factor receptor (EGFR) [54]. Subsequent studies by the same group show that RN-tre specifically accelerates GTP hydrolysis by Rab5 in vitro [32]. Consistently, overexpression of RN-Tre in cultured cells was shown to block Rab-5-dependent EGFR internalization [32]. Mammalian RN-tre was also reported to possess GAP activity towards Rab41 [33] and Rab43 [39]. That reducing the dosage of *Rab5 *or *Rab11 *enhanced the RN-tre-overexpression eye phenotype (Figure 4), together with that loss of *Rab5 *or *Rab11 *caused a R-cell mis-localization phenotype (Figure 5 and 6) identical to that in flies misexpressing RN-Tre (Figure 1, 2, 3), suggests that Rab5 and Rab11 are targets of RN-Tre in *Drosophila*.

While the results from misexpression of RN-Tre provided an entry point for genetic dissection of the role of Rab-mediated vesicular transport in R-cell positioning, the *in vivo *role of RN-Tre remains unknown. Our genetic analysis of available *RN-tre *loss-of-function alleles failed to reveal a defect in R-cell nuclear translocation. There are two possible explanations for the lack of R-cell phenotype in *RN-tre *mutants. First, other RabGAPs may be functionally redundant with RN-Tre and thus compensate for loss of RN-Tre. And second, while Rab-mediated vesicular transport is necessary for maintaining R-cell nuclear positioning, elevated activity of Rabs in *RN-tre *mutants is not sufficient to cause a translocation phenotype. Our current data does not allow us to distinguish among these possibilities.

Rab5, Shi/dynamin and Rab11 have been implicated in regulating vesicular transport in a variety of cellular processes in both vertebrates and invertebrates [41,42,55-57]. Rab5 is required for protein internalization [58], the transport of early endocytic vesicles and their subsequent fusion to the early endosome [59]. Shi/dynamin is also required for the internalization process during the early endocytic pathway (e.g.[57,60,61]). In addition, Shi is required for vesicle recycling and budding during exocytosis [62,63]. Rab11 has been shown to be involved in regulating both endosome recycling (e.g.[48,64,65]) and post-Golgi trafficking of newly synthesized membrane proteins [50]. That loss of Rab5, Shi and Rab11, but not Rab6, caused a similar R-cell mis-localization phenotype suggests strongly that they are involved in a common vesicular transport pathway for maintaining R-cell apical localization during development.

How do Rab5, Shi/dynamin and Rab11 function together to maintain apical localization of R-cell nuclei? One attractive model is that Rab5, Shi/dynamin and Rab11 are involved in a common endocytic-recycling pathway for targeting specific proteins to regulate R-cell positioning. The requirement of the Rab-mediated endocytic-recycling pathway in cell migration has attracted much attention in recent years [66]. For instance, it has been shown that the migration of border cells during *Drosophila *oogenesis requires the endocytosis and subsequent recycling of cell surface receptor tyrosine kinases (RTK) to maintain the polarized localization of RTK signaling at the leading edge [67]. Mammalian cell culture studies also show that the endocytic-recycling pathway is required for maintaining the polarized localization of integrins at the leading edge to control cell migration (e.g. [68-70]). Recent studies also show that Rab-mediated trafficking of E-cadherin plays an important role in regulating cell intercalation [71] and cell polarity during development [72]. Similarly, we speculate that Rab5-Shi-Rab11 may function in R cells to regulate the polarization of specific cell surface proteins, which in turn modulate actin- and microtubule-based cytoskeleton for maintaining apical localization of R-cell nuclei. Such mechanism may also be utilized in mammals to regulate neuronal positioning during brain development. Thus, it will be of interest to determine if the Rab-mediated endocytic-recycling pathway is also required for establishment of tissue architecture in mammalian cerebral cortex, and if this pathway regulates the specific localization of receptor proteins such as APOER2, VLDLR or integrins that have been implicated in mediating cortical neuronal migration in mammals (e.g. [73-76]).

## Conclusion

Mis-regulation of the RabGAP RN-Tre is sufficient to cause a failure of R-cell nuclei to maintain their apical localization in the developing *Drosophila *eye. That RN-Tre interacts genetically with Rab5 and Rab11 suggests that Rab5 and Rab11 are targets of RN-Tre in *Drosophila*. Rab-mediated vesicular transport involving Rab5, Shi/dynamin and Rab11 plays an essential role in maintaining R-cell apical localization in the developing fly eye.

## Competing interests

The authors declare that they have no competing interests.

## Authors' contributions

TH conducted most of experiments, and was involved in writing the manuscript. DvM generated the GSd427 line. LS conducted genetic interaction experiments. YR supervised and wrote the manuscript. All authors read and approve the manuscript.
